# Structural heterogeneity of iron–sulfur cluster scaffold protein IscU: Metamorphic or partially folded?

**DOI:** 10.1063/4.0001219

**Published:** 2026-07-30

**Authors:** Jongbum Na, Minchan Jeong, Jin Hae Kim

**Affiliations:** 1 Department of New Biology, Daegu Gyeongbuk Institute of Science and Technology (DGIST), Daegu 42988, Republic of Korea; 2 New Biology Research Center, Daegu Gyeongbuk Institute of Science and Technology (DGIST), Daegu 42988, Republic of Korea

## Abstract

The scaffold protein IscU plays a central role in iron–sulfur (Fe–S) cluster assembly and transfer. Despite extensive structural and biochemical research, the conformational nature of IscU remains unclear. IscU has been proposed as a metamorphic protein that interconverts between structured (S) and disordered (D) states. However, its conformational heterogeneity has often been interpreted as a consequence of intrinsic marginal stability, with the D-state described as an unfolded and functionally irrelevant state. These contrasting views highlight the fundamental challenge in defining the structural identity of IscU. In this review, we provide a comprehensive overview of the structural heterogeneity of IscU and examine how intrinsic and extrinsic factors shape the conformational equilibrium between the S- and D-states. Furthermore, we consider how these conflicting interpretations could be reconciled within an ensemble-based framework. In addition, we highlight promising approaches for characterizing the structurally elusive D-state, including high-pressure nuclear magnetic resonance, single-molecule Förster resonance energy transfer, native ion mobility mass spectrometry, and artificial intelligence-based structural analysis, all of which provide new opportunities for probing low-populated and transient conformational states. Collectively, these perspectives suggest that the functional behavior of IscU is closely linked to its conformational heterogeneity and that a conformational landscape view may provide a more integrated understanding of its role in Fe–S cluster biosynthesis.

## I. INTRODUCTION

Proteins are not static entities but inherently dynamic molecules.^1^ The notable dynamic and adaptable features of living organisms originate, at least in part, from the versatility of biomolecules, particularly proteins, the molecular machinery that mediates most physiological processes.^2,3^ Recently, numerous studies have reported a variety of dynamic characteristics of proteins, ranging from simple disordered movement of tails or entropic linkers to intrinsically disordered proteins (IDPs) and intrinsically disordered region (IDR)-containing proteins.^4^ In most cases, these structural dynamics are not the result of failure to secure sufficient stability; rather, they reflect a sophisticated and intentional design to balance versatile functionality and durability.^5,6^ For instance, Hsp70 molecular chaperones contain an evolutionarily conserved disordered linker between their nucleotide- and substrate-binding domains. This linker helps to coordinate the communication between the two domains,^7,8^ illustrating how IDR dynamics can contribute to protein regulation. Another example is the TAR DNA-binding protein 43 kDa (TDP-43), which contains multiple IDRs. Its C-terminal low-complexity region is involved in biomolecular condensate formation, and altered condensate behavior has been associated with TDP-43 pathology.^9,10^ Thus, TDP-43 serves as a prominent example of how IDR-mediated dynamic interactions are linked to normal cellular functions and disease.

Notably, metamorphic proteins exhibit striking conformational dynamics, interconverting between multiple distinct conformations under physiological conditions. In several metamorphic proteins, the interconversion between distinct conformational states is directly related to physiological functions. For instance, the circadian clock protein KaiB slowly interconverts between the ground-state and fold-switched conformations, thereby regulating circadian timing by modulating KaiC phosphorylation.^11^ Similarly, the spindle checkpoint protein Mad2 exists in equilibrium between the open and closed conformations, and only closed Mad2 can bind to Cdc20 to inhibit anaphase progression during mitosis.^12^ In addition, the metamorphic protein lymphotactin (XCL1) adopts two structurally distinct states with separate biological functions: the monomeric Ltn10 state functions in chemokine receptor signaling, whereas the dimeric Ltn40 state promotes glycosaminoglycan binding.^13^ These examples illustrate that interconversion between metamorphic states serves as a key mechanism for functional switching across diverse biological processes.

However, the conformational nature of IscU, an iron–sulfur (Fe–S) cluster scaffold protein that was also proposed to be a metamorphic protein, remains controversial. Nuclear magnetic resonance (NMR)-based investigations have identified the conformational heterogeneity of this protein, the well-folded structured state (S-state) and the dynamically disordered state (D-state), which interconverts under physiological conditions.^14^ Markley *et al.* interpreted this as maximizing the functional multivalency and proposed that both conformational states have physiological relevance.^15^ However, Pastore *et al.* characterized this as a structural imperfection, suggesting that additional ligands, such as zinc, are required to fulfill its physiological roles.^16^ In this review, we summarize the current progress in characterizing the structural and functional versatility of IscU and discuss how these seemingly conflicting interpretations may be reconciled. Moreover, we propose novel and promising methodologies that may contribute to bridging the gap toward a unified understanding of IscU's structural and functional attributes.

## II. RECENT PROGRESSES IN CHARACTERIZING THE STRUCTURAL HETEROGENEITY OF ISCU

### A. Fe–S clusters and mechanisms of their biogenesis

Fe–S clusters are among the most highly conserved cofactors in living organisms and play crucial roles in maintaining protein structure as well as enzymatic reactivity.^17^ Fe–S clusters are required for a wide range of physiological pathways, including the tricarboxylic acid cycle, heme biosynthesis, and iron homeostasis.^18^ Accordingly, defects in the Fe–S cluster assembly or transfer are associated with severe diseases in humans, including Parkinson's disease and Friedreich's ataxia.^18–20^

**FIG. 1.**
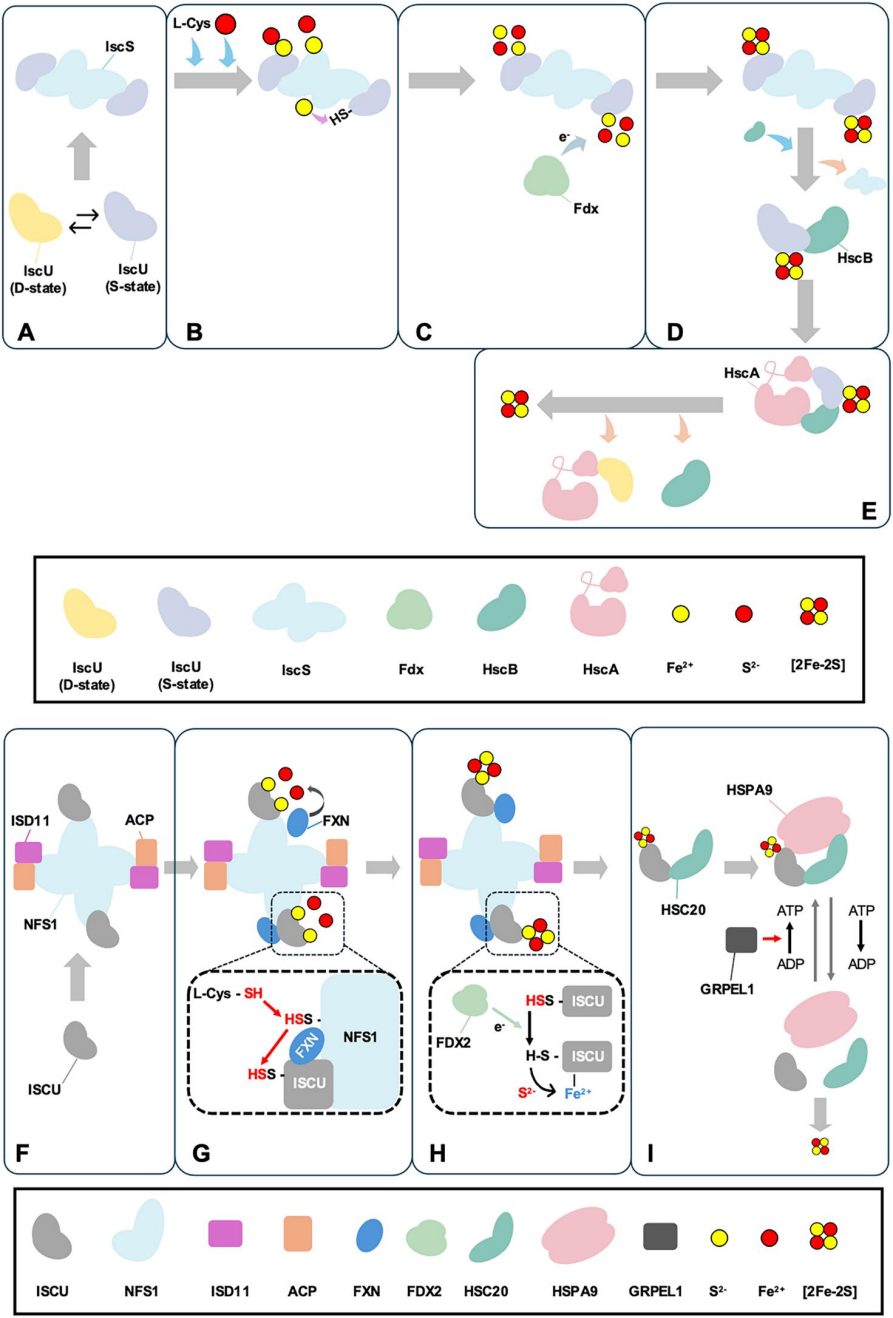
Overview of the Fe–S cluster biosynthesis mechanism. (a)–(e) [2Fe-2S] cluster biosynthesis in *E. coli*. (a) IscU assembles with IscS to form a 1:1 heterotetrameric complex. (b) IscS provides sulfur derived from L-cysteine to IscU. (c) Fdx (ferredoxin) transfers electrons required for [2Fe-2S] cluster assembly on IscU. (d) The [2Fe-2S] cluster-IscU complex dissociates from IscS and subsequently associates with HscB (Hsp40 cochaperone family) and HscA (Hsp70 chaperone family). (e) HscA facilitates the transfer of the [2Fe-2S] cluster from IscU to recipient proteins. Figure was modified from Ref. 78. (f)–(i) [2Fe-2S] cluster biosynthesis mechanism in human mitochondria. (f) The NFS1-ISD11-ACP (NIA) complex assembles with ISCU. (g) The NIA complex provides sulfur derived from L-cysteine, while FXN (frataxin) enhances catalytic activity, stabilizes the complex conformation within the ISCU-NIA complex. (h) FDX2 (ferredoxin) transfers electrons required for [2Fe-2S] cluster assembly on ISCU. (i) The [2Fe-2S] cluster-ISCU complex dissociates from the NIA complex and subsequently interacts with HSC20 (Hsp40 cochaperone family) and HSPA9 (Hsp70 chaperone family) to mediate cluster transfer to recipient proteins. GRPEL1 functions as a nucleotide exchange factor that regulates the HSPA9 chaperone cycle.

Several mechanisms are responsible for Fe–S cluster biogenesis.^21^ Among these, the Fe–S cluster (ISC) system is considered to be one of the most evolutionarily conserved^22^ and serves as the primary machinery for the Fe–S cluster assembly in both prokaryotes and eukaryotic mitochondria^21^ (Fig. 1). The *Escherichia coli* ISC system is an established model for studying Fe–S cluster biogenesis. In *E. coli*, genes involved in the ISC pathway are organized in an operon-like gene cluster that includes the transcriptional regulator IscR, cysteine desulfurase IscS, scaffold protein IscU, chaperone/co-chaperone pair HscA/HscB, and ferredoxin (Fdx)^23^ [Figs. 1(a)–1(e)]. Although the mitochondrial ISC system in eukaryotes shares core components with the prokaryotic system, its composition and structural organization are more diversified^24^ [Figs. 1(f)–1(i)]. Still, IscU serves as a central hub of the ISC machinery by coordinating interactions with partner proteins and facilitating the Fe–S cluster assembly and transfer.^24^

The components of the ISC machinery work in a concerted manner to mediate the Fe–S cluster assembly. First, IscS and IscU associate to form a heterotetrameric complex.^25,26^ IscS mobilizes sulfur from an L-cysteine and transfers it to IscU,^27^ whereas a ferrous ion (Fe^2+^) is delivered to IscU by an iron donor protein.^28^ The electrons required for the [2Fe–2S] cluster assembly are supplied by ferredoxin.^29^ The resulting [2Fe–2S]-bound IscU is recognized by HscB, which delivers it to HscA.^30^ Upon binding to HscA, HscB dissociates from the complex and the D-state of IscU is stabilized, facilitating the transfer of the [2Fe–2S] cluster from IscU to recipient apo-proteins^31^ (Fig. 1).

### B. Structural conservation and heterogeneity of IscU

**FIG. 2.**
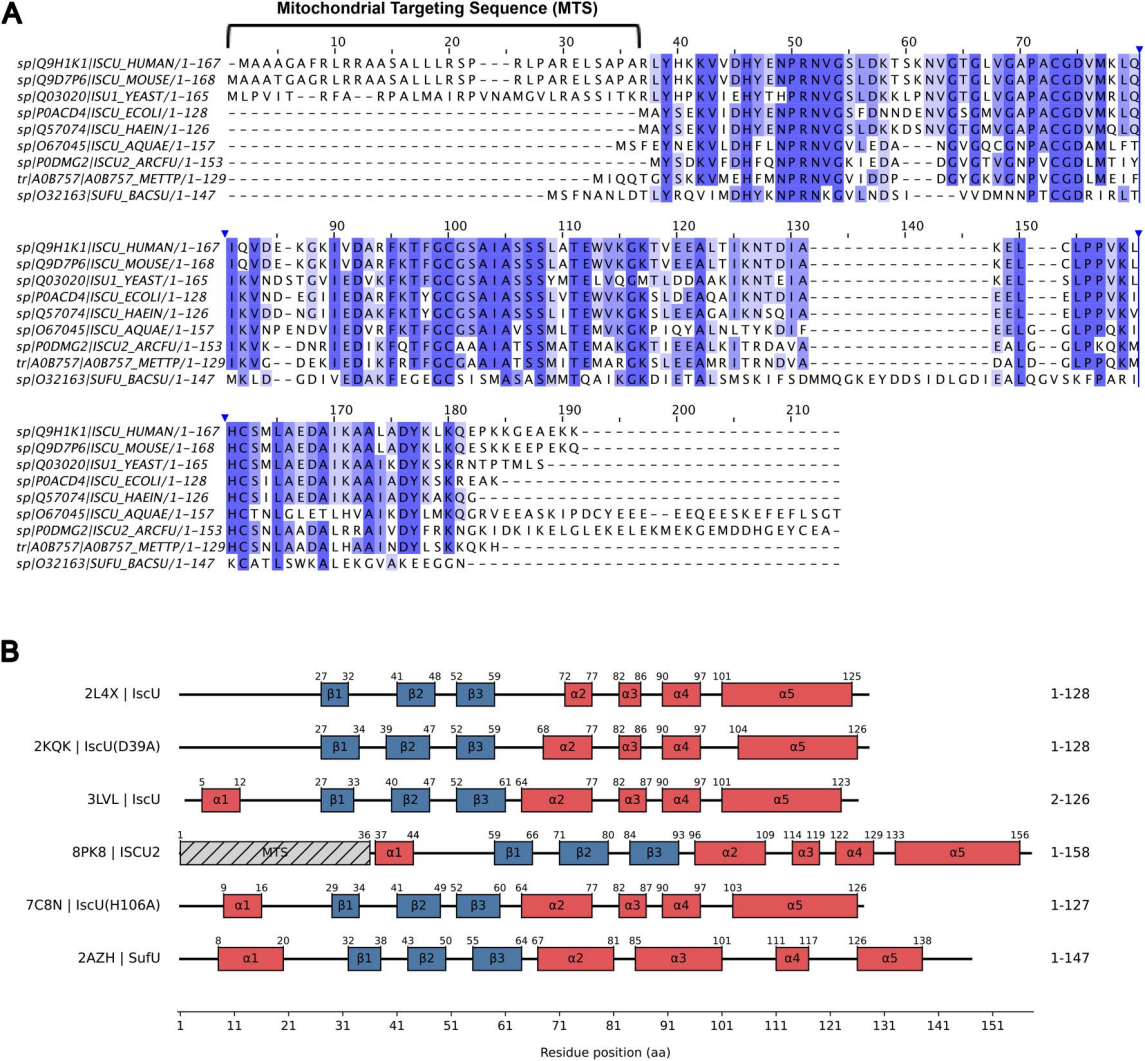
Amino acid sequence and secondary structure alignments of IscU homologs. (a) Sequence alignment of IscU homologs from diverse species, including HUMAN (UniProt: Q9H1K1; *Homo sapiens*), MOUSE (UniProt: Q9D7P6; *Mus musculus*), YEAST (UniProt: Q03020; *Saccharomyces cerevisiae*), HAEIN (UniProt: Q57074; *Haemophilus influenzae*), AQUAE (UniProt: O67045; *Aquifex aeolicus*), ARCFU (UniProt: P0DMG2; *Archaeoglobus fulgidus*), METTP (UniProt: A0B757; *Methanothrix thermoacetophila*), and SUF_BACSU (UniProt: O32163; *Bacillus subtilis*). The blue shading indicates sequence identity, with darker shades representing higher conservation. Eukaryotic IscU homologs contain a mitochondrial targeting sequence (MTS). (b) Secondary structure alignment of IscU homologs based on structure deposited in PDB. Red boxes indicate α-helices, blue boxes indicate β-strands, and hatched boxes indicate the MTS region.

**FIG. 3.**
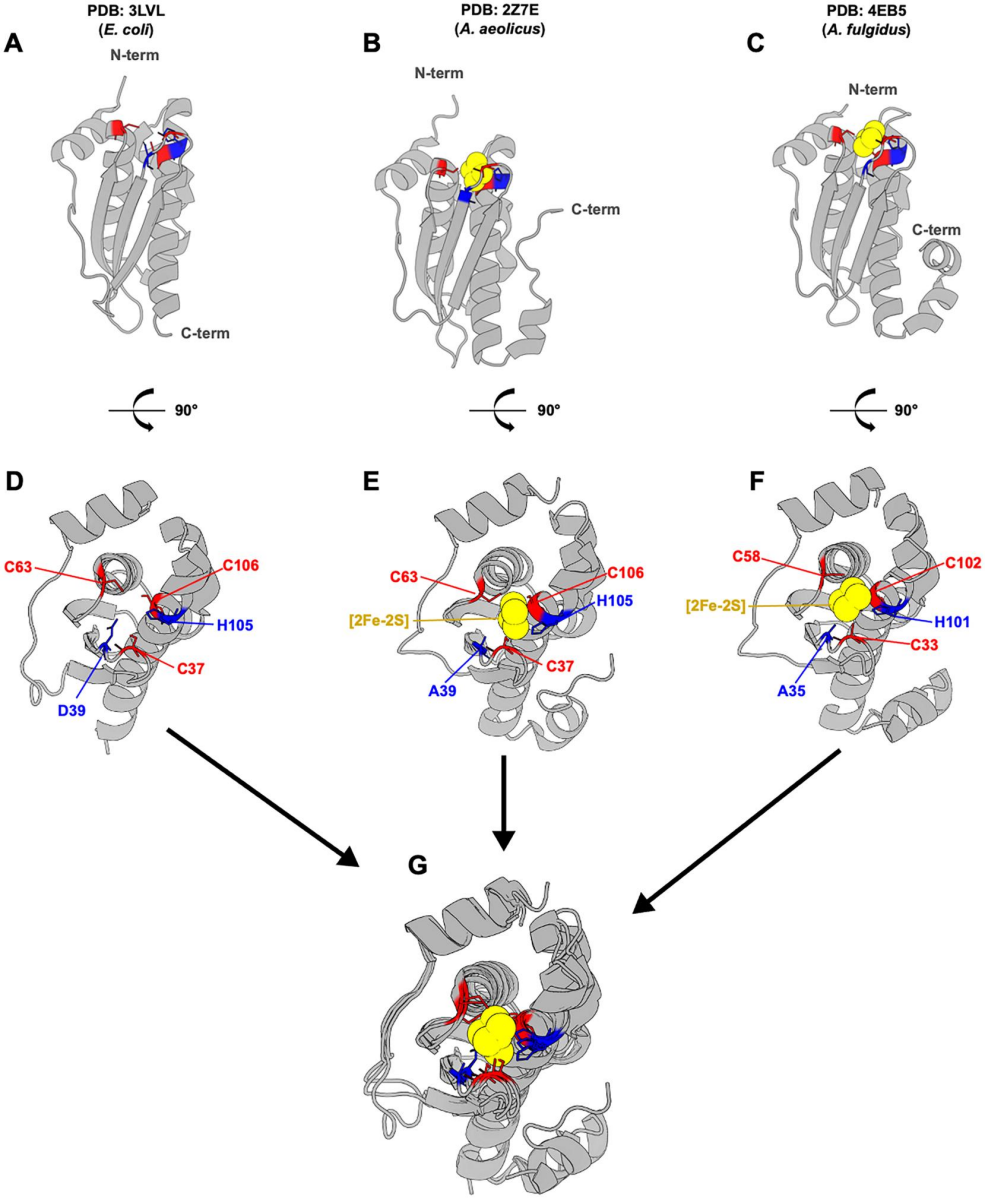
Metal-binding sites of IscU homologs. (a) and (d) The side and top views of the *E. coli* IscU structure from the cluster-free IscU-IscS complex (PDB: 3LVL). (b) and (e) The side and top views of the *A. aeolicus* homo-trimeric IscU containing a [2Fe-2S] cluster (PDB: 2Z7E). In this structure, the residue corresponding to D39 in *E. coli* IscU is replaced by alanine (A39). (c) and (f) The side and top views of the *A. fulgidus* IscU-IscS complex containing a [2Fe-2S] cluster (PDB: 4EB5). In this structure, the residue corresponding to D39 in *E. coli* IscU Vis replaced by alanine (A35). The three invariant cysteine residues are shown in red. Conserved histidine and aspartate/alanine residues are shown in blue. The [2Fe-2S] cluster is shown as yellow spheres. (g) Superposition of the top-view structures highlighting overall structural similarity with local diversity in the metal-binding environments among IscU homologs.

In solution, the S-state of IscU adopts an ellipsoid-like structure composed of three β-strands and five α-helices (Figs. 2 and 3). At one pole of the protein, three conserved cysteine residues (C37, C63, and C106 in *E. coli* IscU), along with one aspartate residue (D39 in *E. coli* IscU), are clustered to form the active-site region, which plays a central role in [2Fe–2S] cluster assembly and transfer^32^ (Fig. 3). Consistent with its essential function as a scaffold protein, IscU and its homologs exhibit high sequence conservation across species^33^ [Fig. 2(a)], indicating the preservation of a common structural framework. Accordingly, experimentally determined structures of IscU homologs display highly similar global folds with low backbone RMSD values (Table I and Fig. 3).

**TABLE I.** Representative structural features of IscU homologs.

Organism	Protein	PDB ID	Year	Method	Structural features	Backbone RMSD (Å; relative to 2L4X)	References
*E. coli*	IscU	2L4X	2012	NMR	WT apo-IscU, S-state model	0	39
*E. coli*	IscU	2KQK	2012	NMR	D39A mutant, S-state to >95%	1.128	39
*E. coli*	IscU	3LVL	2010	X ray	IscS-bound	1.018	25
*H. sapiens*	ISCU	5WLW	2017	X ray	Bound to NFS1-ISD11-ACP	1.001	40
*H. sapiens*	ISCU	8ZZF	2018	Hybrid	Bound to FXN-NFS1-ISD11-ACP (complete active complex)	1.001	79
*H. sapiens*	ISCU	8PK8	2024	Cryo-EM	Full mitochondrial ISC complex during persulfide transfer	0.856	41
*S. cerevisiae*	Isu1	5T0V	2016	Cryo-EM	Isu1-Yfh1 (frataxin) 48-mer complex	3.171	50
*S. cerevisiae*	Isu1	5TRE	2016	Cryo-EM	Isu1-Yfh1 (frataxin) 48-mer complex	2.75	80
*M. musculus*	IscU	1WFZ	2004	NMR	Zn^2+^-bound	0.866	81
*H. influenzae*	IscU	1R9P	2003	NMR	Zn^2+^-bound	4.295	38
*A. aeolicus*	IscU	2Z7E	2007	X ray	[2Fe-2S] cluster-bound	2.034	43
*A. fulgidus*	IscU	4EB5	2012	X ray	IscS-bound	1.2	26
*M. thermoacetophila*	IscU	7C8N	2021	X ray	H106A dimer with [2Fe-2S] cluster	1.542	44
*B. subtilis*	SufU	2AZH	2005	NMR	Zn^2+^-bound SufU (IscU ortholog in the SUF system)	4.017	82

Although the eukaryotic mitochondrial ortholog ISCU (Isu1 in yeast) shares strong structural and functional similarities with the prokaryotic IscU,^34^ several important distinctions remain (Table I). ISCU contains an additional N-terminal mitochondrial targeting sequence that is removed upon mitochondrial import^35^ (Fig. 2). In addition, the Fe–S cluster assembly in eukaryotes is mediated by a more elaborate molecular machinery than that in prokaryotes. Human ISCU functions alongside NFS1, ISD11, ACP, and FXN, whereas *E. coli* IscU primarily operates in conjunction with the cysteine desulfurase IscS^24,34^ (Fig. 1).

**FIG. 4.**
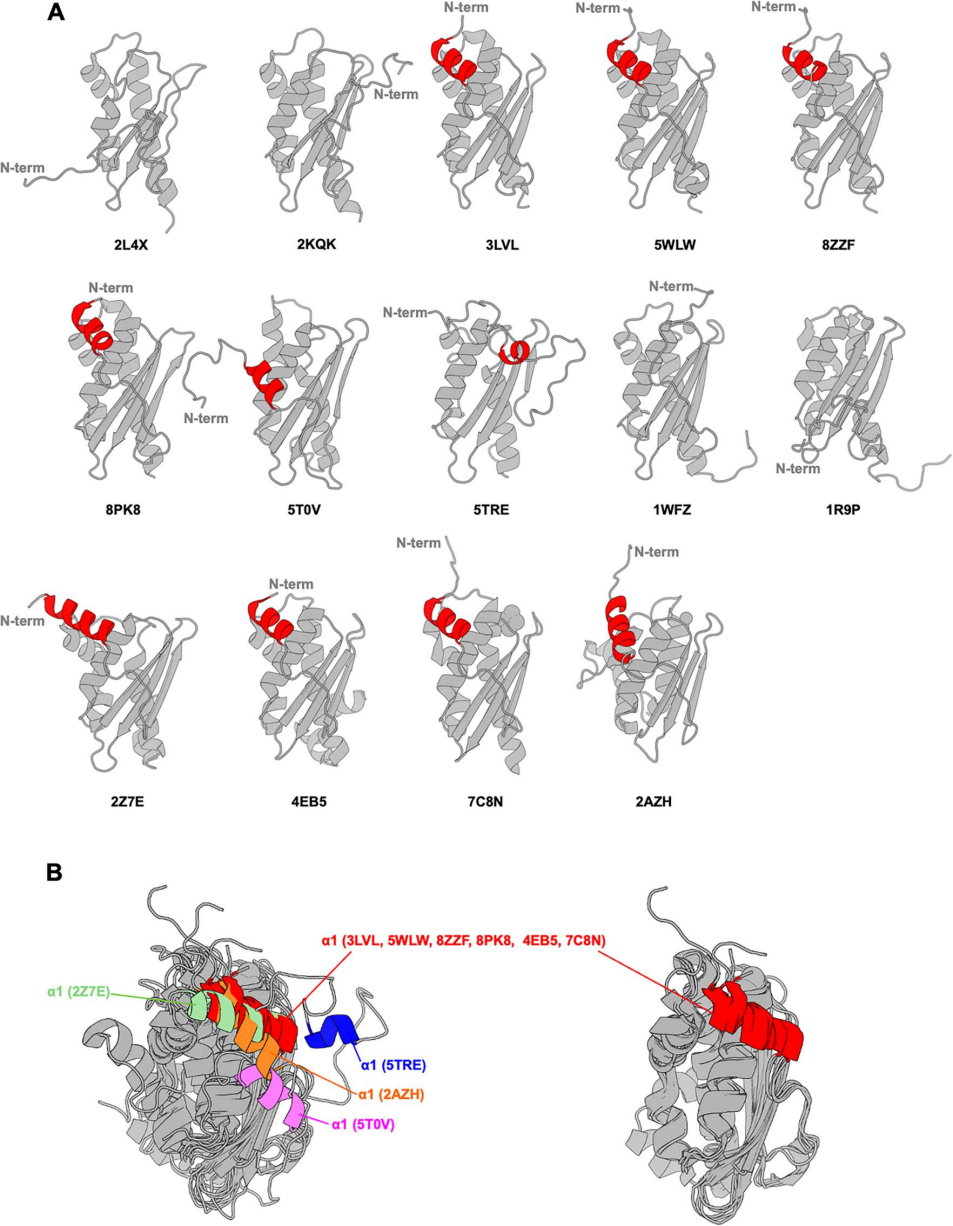
Structural heterogeneity of IscU. (a) Representative structural models of IscU homologs (PDB 2L4X, 2KQK, 3LVL, 5WLW, 8ZZF, 8PK8, 5T0V, 5TRE, 1WFZ, 1R9P, 2Z7E, 4EB5, 7C8N, and 2AZH; see Table I). The α-helices (α1) in the N-terminal loop region are highlighted in red. Models lacking α-helical structure in the corresponding positions were left uncolored (2L4X, 2KQK, 1WFZ, and 1R9P). (b) Superposition of IscU structures that form α1 in the N-terminal loop region. The α1 motif in 3LVL, 5WLW, 8ZZF, 8PK8, 4EB5, and 7C8N are colored red, where they share similar spatial positions. Other models, displaying different localization of α1, are colored green (2Z7E), blue (5TRE), orange (2AZH), and pink (5T0V).

The IscU homologs also exhibit structural heterogeneity (Fig. 4). One of the most prominent examples is the N-terminal loop, a major source of conformational variability.^36^ Structural ensembles obtained through solution NMR spectroscopy demonstrated that this region remains highly flexible and samples multiple conformations in solution^37–39^ (Fig. 4). In contrast, structures determined in complex with partner proteins or within homo-oligomeric assemblies, typically through x-ray crystallography or cryo-electron microscopy (cryo-EM), often show that this N-terminal loop adopts an α-helical conformation, referred to as α1.^25,40,41^ These observations suggest that α1 helix formation is primarily influenced by environmental factors, as evidenced by the distinct conformations adopted by *E. coli* IscU under different experimental conditions, including apo solution NMR structure (PDB: 2L4X) and partner-bound crystal structure (PDB: 3LVL) [Fig. 4(a)]. Notably, the α1 helix exhibits a conserved spatial arrangement across these structures [Fig. 4(b)]. The primary exception is found in the yeast structures (PDB: 5TRE and 5T0V), where the α1 helix adopts a different orientation, potentially reflecting the combined effects of oligomeric assembly and local sequence divergence [Fig. 4(b) and Table I].

This structural tendency is consistent with the presence of conserved residues in the α1 region, including a conserved tyrosine residue (residue Y3 in *E. coli* IscU) that contributes to interactions with the cysteine desulfurase IscS^42^ [Fig. 2(a)]. These observations suggest that the N-terminal region undergoes binding-coupled folding, shifting from a flexible conformational ensemble in solution to a more ordered helical structure upon complex formation.^36^ However, in the absence of partner protein binding, the N-terminal loop remains dynamically disordered, as observed in solution NMR studies.^38,39^ Importantly, this local structural heterogeneity is not merely a passive feature of a flexible loop; rather, it appears to be closely linked to the global conformational equilibrium of IscU,^36^ which is discussed in Sec. II C.

IscU also exhibits significant flexibility in its cluster-binding site (Fig. 3). Previous studies have consistently shown distinct Fe–S cluster coordination patterns under different experimental conditions or upon mutations introduced in this region.^14,43,44^ IscU coordinates the 2Fe–2S cluster primarily through three conserved cysteine residues (C37, C63, and C106 in *E. coli*), whereas an aspartate residue (D39 in *E. coli*) often serves as the fourth coordinating ligand.^45^ However, one study proposed that this fourth coordination position for the Fe–S cluster may exist in a “tug-of-war” state between residues D39 and H105.^32^ Because these residues are located close to the cluster-binding site, they may exert conflicting effects on the coordination environment, thereby contributing to a relatively labile coordination environment.^32^ Intriguingly, in a crystallographic study of *Methanothrix thermoacetophila* 2Fe–2S cluster-bound IscU dimer, where the aspartate and histidine residues were both substituted with alanine, the 2Fe–2S cluster could still be sustained by three cysteines from one subunit and one cysteine from the other, revealing the intrinsic adaptability of the IscU cluster-binding site (PDB 7C8O).^46^ This structural flexibility and adaptability are consistent with the functional requirement that IscU mediates both the Fe–S cluster assembly and its subsequent transfer.

The structural heterogeneity of IscU is not limited to local flexibility. Cowan *et al.* and Bertini *et al.* reported that *Thermotoga maritima* IscU exhibits molten globule-like characteristics. However, because its conformational exchange is slower than that typically observed for conventional molten globules, they proposed that IscU represents a more complex system composed of multiple discrete conformers.^47,48^ In addition to its conformational diversity, multiple oligomeric states of IscU have also been reported. Although *E. coli* IscU is predominantly monomeric under physiological conditions, its oligomeric form has been identified in several species. For instance, yeast Isu1 was shown to form a 24-mer oligomeric complex with frataxin (Yfh1)^49,50^ (Table I). IscU has also been observed in homodimeric and homotrimeric forms in *Aquifex aeolicus* and *T. maritima*, respectively, during the intermediate stages following [2Fe–2S] cluster assembly and transfer^43,44^ (Table I). These observations indicate that IscU heterogeneity is manifested at multiple structural levels, ranging from local conformational flexibility to global state transitions and higher-order oligomeric assemblies.

### C. Core of structural heterogeneity: The metamorphic model

Advancing one step further, Markley *et al.* discovered even greater flexibility across the entire IscU molecule, leading them to propose IscU as a metamorphic protein.^15^ *E. coli* IscU exists in equilibrium between the S- and D-states, with populations of approximately 80% and 20%, respectively, under ambient conditions.^39^ They also identified several intrinsic structural factors that regulate equilibrium. One important factor is the cis–trans isomerization of the conserved proline residues (P14 and P101 in *E. coli* IscU).^51^ Under D-state-stabilizing conditions, these proline residues undergo trans-to-cis isomerization, generating cis-peptide bonds that are thermodynamically less favorable than their trans form.^51^ These observations led Markley *et al.* to propose that the D-state retains ordered structural elements capable of stabilizing the energetically unfavorable cis peptide bonds, instead of representing a completely disordered conformation.^51^ Because proline isomerization involves a substantially higher energy barrier than typical backbone conformational rearrangements, it represents one of the key factors governing the relatively slow interconversion between the S- and D-states (S → D, 0.77 s^−1^ and D → S, 2.0 s^−1^).^37^ In addition to proline isomerization, our recent study has highlighted the role of the N-terminal IDR in modulating this equilibrium.^36^ Specifically, increased helical propensity (α1 motif; residues S4-N13 in *E. coli* IscU) within the N-terminal loop stabilizes the S-state population, thereby influencing the balance between the two conformational states.

In addition to intrinsic determinants, the conformational equilibrium of IscU is further modulated by extrinsic factors, including interactions with partner proteins, such as IscS, HscA, and HscB, as well as by metal ions and cofactors such as Zn^2+^, Fe^2+^, and the [2Fe–2S] cluster, which help explain the functional versatility of IscU.^31,37,38,43^ Notably, these intermolecular interactions occur with an evident preference for one conformation over the other. Binding to HscB and metal ions generally shifts the equilibrium toward the S-state,^14,31^ whereas HscA preferentially recognizes and stabilizes the D-state.^31^ Consistent with this, HscA shows an increased affinity for the D-state-stabilizing mutants of IscU.^36^ Equilibrium is also sensitive to environmental conditions such as pH and temperature. For instance, the D-state of IscU dominates at low pH and at both high and low temperatures.^52^

Collectively, these findings support a model in which the metamorphic equilibrium of IscU functions as a structural switching mechanism that underlies its regulatory role in Fe–S cluster biosynthesis, thereby contributing to both the efficiency and fidelity of the process. Importantly, this regulatory mechanism is conserved across species. Human mitochondrial ISCU undergoes comparable equilibrium shifts in response to partner–protein interactions and metal binding, including interactions with NFS1, the human ortholog of *E. coli* IscS.^53^

### D. Mutational control of IscU's metamorphic behavior

Studies on IscU variants have provided important insights into the molecular mechanisms underlying the metamorphic equilibrium of IscU. Several variants, even those with a single amino acid substitution, exhibited significant equilibrium perturbations, indicating that the amino acid sequence of IscU is fine-tuned to maintain a delicate balance between the two conformations. Markley *et al.* systematically investigated a series of *E. coli* IscU mutations (K89A, N90A, N90D, S107A, and E111A) to examine how the stability of the structured core, formed by the α3–α4 and α5 helices, influences the metamorphic equilibrium.^37^ Among these variants, K89A and N90D shifted the equilibrium toward the D-state by destabilizing the structured core, whereas N90A, S107A, and E111A shifted the equilibrium toward the S-state, likely by strengthening packing interactions between the α3, α4, and α5 helices^37^ (Table II).

**TABLE II.** Effects of mutations on the metamorphic equilibrium and functional property of IscU.

Mutation on IscU	Organism	Changes in metamorphic equilibrium	Structural and functional effects	PDB and References
Y3A	*E. coli*	D-state ↑	Abolishes i*n vivo* function; disrupts functional interface with IscS	36 and 42
Y3M, Y3W	*E. coli*	S-state ↑	Preserve hydrophobic packing at the N-terminus	36
I8K	*E. coli*	S-state ↑	Artificial stabilization of the transient ⍺1 helix	
V7G, D9G, Y11A, Y11P, R15A	*E. coli*	D-state ↑	Disrupt local interaction network required for ⍺1 stabilization	
D39A, D39V	*E. coli*	S-state ↑	Increases Fe–S cluster stability; used as a “folded state” surrogate	2KQK^39^
K89A, N90D	*E. coli*	D-state ↑	Conformational tuning residues in the ⍺4-⍺5/C-terminal regions that modulate the S/D equilibrium	37
N90A, S107A, E111A	*E. coli*	S-state ↑		37
G50E	*H. sapiens*	n.d.^a^	Recessive missense mutation causing ISCU-myopathy disease; weakens interaction with NFS1/HSCB and impairs Fe–S cluster synthesis	58 and 60
G96V	*H. sapiens*	n.d.	Dominant missense mutation causing muscle weakness; detrimental effect on Fe–S enzyme	59
D37A	*S. pombe*	n.d.	Homologous to *E. coli* D39A; stabilizes the cluster; D39 is required for efficient cluster release	83
D43A	*B. subtilis*	n.d.	Homologous to *E. coli* D39A; stabilizes the cluster, allowing aerobic purification of holo-SufU	84
H106A	*M. thermoacetophila*	n.d.	Stabilizes the cluster without affecting the dimeric state	7C8N^44^
D40A/H106A (double mutation)	*M. thermoacetophila*	n.d.	Probes the role of D40 in cluster coordination	7C8O^46^

^a^ n.d.: not determined.

In addition, the Fe–S cluster-coordinating pole of the IscU ellipsoid also plays an important role in modulating the conformational equilibrium. Among the residues within this region, D39 has been extensively studied in multiple systems.^54–56^ D39A substitution has been shown to enhance the structural stability of IscU and favor a more competent conformation for Fe–S cluster coordination.^54–56^ Consistent with this finding, Kim *et al.* reported that the D39A mutation shifts the metamorphic equilibrium toward the S-state (Table II).^14^ These observations indicate that the stabilization of the cluster-coordinating region is closely linked to the broader conformational landscape of IscU.^14,57^

**FIG. 5.**
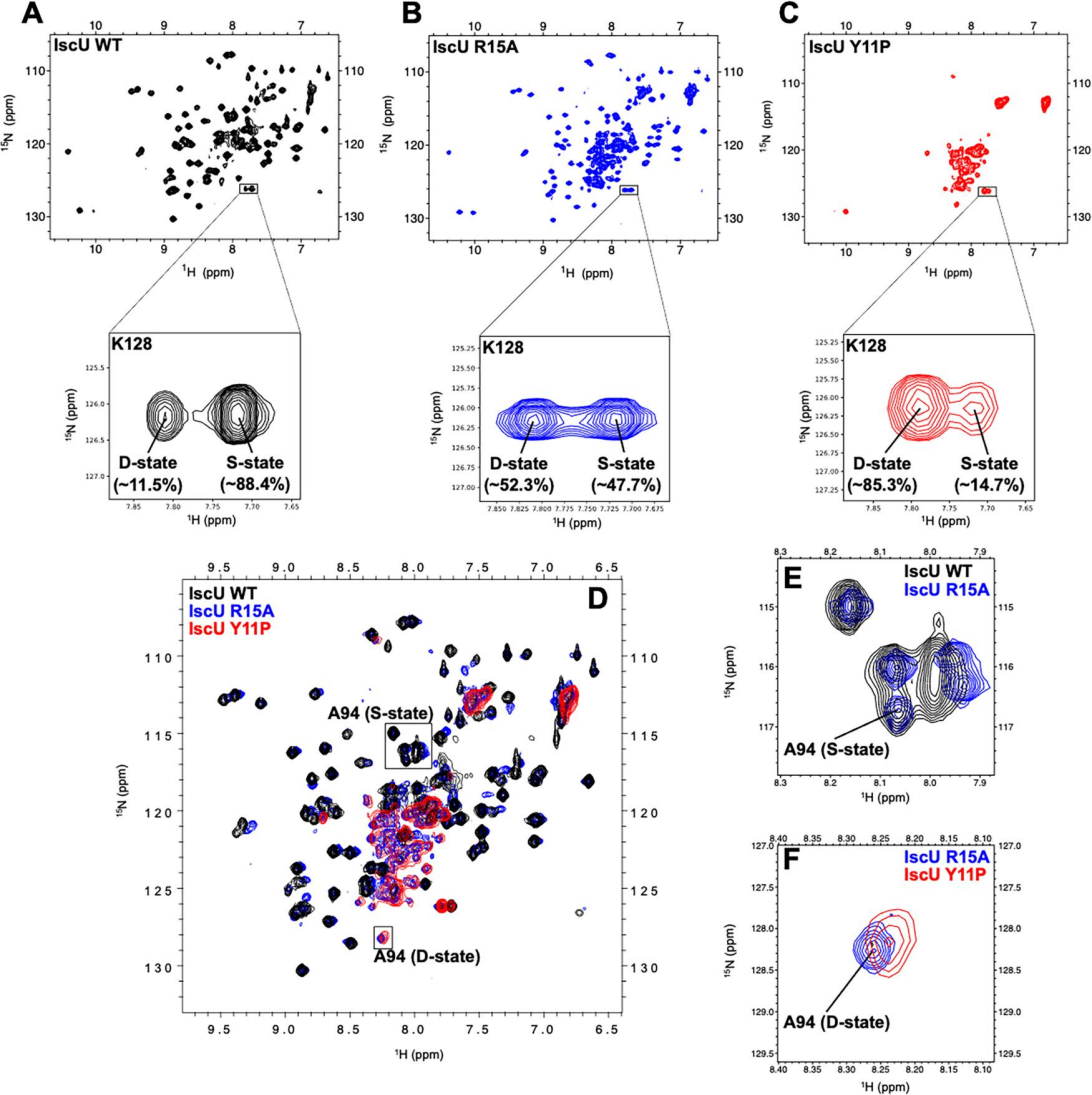
Metamorphic behavior of *E. coli* IscU observed by NMR. (a)–(c) Representative ^1^H-^15^N HSQC spectra of IscU variants illustrating previously reported S- and D-state populations for IscU WT (black; ∼11.6% D-state), R15A (blue; ∼52.3% D-state), and Y11P (red; ∼85.3% D-state).^36^ Population were estimated based on peak intensities of residue K128.^39^ Spectra and population values were adapted from previous work.^36^ (d)–(f) Overlaid ^1^H-^15^N HSQC spectra of WT, R15A, and Y11P. (d) Full spectral comparison. (e) Expanded view of the S-state peak for the residue A94 in WT and R15A. (f) Expanded view of the D-state peak for the residue A94 in R15A and Y11P.

As discussed in Section II C, the N-terminal region of IscU has emerged as a critical determinant of its metamorphic behavior. Previous studies have shown that substitutions at Y3 affect interactions with partner proteins, including the cysteine desulfurase IscS, thereby influencing the Fe–S cluster assembly in *E. coli*, as supported through *in vivo* complementation assays.^42^ Extending these observations, we recently showed that the side chain bulkiness at this position plays a key role in tuning the metamorphic equilibrium. Specifically, the Y3A mutation shifted the equilibrium toward the D-state, whereas Y3W shifted it toward the S-state, indicating that the side chain size contributes to conformational tuning^36^ (Table II). In addition, helical propensity within the α1 motif (residues S4–N13) further modulates this equilibrium. The I8K variant, which enhances α-helical propensity, shifted the equilibrium toward the S-state; conversely, mutations predicted to reduce helical propensity, such as V7G, D9G, Y11A, Y11P, and R15A, shifted the equilibrium toward the D-state^36^ (Fig. 5). Because the N-terminal region of IscU participates as a critical interface in oligomerization or in the interaction with cysteine desulfurase, these findings suggest that this region likely acts as a regulatory hub modulating the global conformational equilibrium of IscU in response to various interactions.

Mutations identified in eukaryotic systems highlight the functional significance of this equilibrium. For instance, human ISCU variants, such as G50E and G96V, have been associated with myopathy^58,59^ (Table II). In particular, the G50E variant has been reported to disrupt interactions with partner proteins, including NFS1 and HSCB, thereby impairing Fe–S cluster biosynthesis.^60^ Although the direct effects of these variants on metamorphic equilibrium have not yet been established, they may reshape the underlying conformational landscape of ISCU, thereby interfering with its functional cycling.

### E. The debate: Metamorphosis vs marginal stability

In contrast to the model of IscU as a metamorphic protein, Pastore *et al.* provided substantial evidence suggesting that IscU is a “marginally stable” protein.^16,61^ In this context, the D-state of IscU is not a specific functional state but instead a by-product of the failure to form a stable structure. Iannuzzi *et al.* demonstrated the importance of zinc ions in the thermal stability of IscU and Fe–S cluster formation.^16^ Furthermore, they observed that low ionic strength (salt-free condition) decreased the structural stability of IscU. Given that under physiological conditions, zinc ions are ubiquitous and the ionic strength is not low, they argued that the D-state of IscU in the experimental conditions is simply an artifact arising from the absence of zinc ions or the low ionic strength of the buffer.^16^ Yan *et al.* narrowed their suggestion to the D-state of IscU.^61^ They challenged the previously proposed role of D-state IscU in the formation of the IscU:IscS complex by Markley *et al.*^37^ They further demonstrated that the state that participates in complex formation is the S-state of IscU, consistent with a previously reported x-ray crystallography model of the complex.^25^ Yan *et al.* supported this argument by suggesting that the D-state of IscU is a partially unfolded state resulting from marginal instability^57^ originating from the electrostatically frustrated region in IscU, where conserved cysteine residues are clustered. They emphasized the roles of the cysteine residues and D39 in the structural stability of IscU.^57^

Two contrasting conclusions regarding the dynamic behavior of IscU may arise, at least in part, from the unique structural properties of IscU, which are distinct from those of canonical metamorphic proteins. In representative metamorphic proteins such as Mad2 and XCL1, both interconverting states are well structured and functionally competent.^12,13^ IscU, however, does not fit this pattern; although the S-state is relatively well structured, as evidenced by crystal structures, the D-state displays features reminiscent of an IDP, as observed through solution NMR (Fig. 5). The strong dependence of the D-state of IscU on buffer conditions and the presence/absence of cofactors is also consistent with the behavior commonly observed in IDPs.^14,16,31,37,62^ Therefore, the central issue is whether this D-state reflects an intrinsic structural property of IscU or is an artifact induced by non-physiological experimental conditions.

The origin of disorderedness of IscU can be understood through a framework that partially reconciles these two views. As proposed by Pastore *et al.*, the electrostatic gates of IscU may lower the energy barrier between the S- and D-states, thereby facilitating interconversion. Simultaneously, the proline isomerization mechanism (proposed by Markley *et al.*) and/or the order-disorder transition of the N-terminal α1 motif (proposed by us) may act as a direct trigger for this transition.^51^ The presence of Zn^2+^, Fe–S clusters, and partner proteins can further shift the conformational equilibrium by stabilizing one state over the other.^14,38,39,43^ In this view, the structural heterogeneity of IscU is best interpreted not as the consequence of a single determinant, but as the result of an interplay between intrinsic conformational flexibility and environment-dependent stabilization.

As discussed above, the structural state of IscU upon binding to partner proteins or cofactors such as Zn^2+^ has been central to the long-standing disagreement between Markley *et al.* and Pastore *et al.* One of the major reasons for this controversy is the size limitations of NMR spectroscopy. When IscU forms a complex with partner proteins, particularly IscS, the molecular weight of the complex exceeds 40 kDa, which is close to or beyond the practical size limit for conventional NMR analysis. Pastore *et al.* addressed this challenge using methyl labeling and TROSY-based experiments and provided convincing evidence that IscS-bound IscU maintains a structured conformation.^61^

In 2022, Lin *et al.* further investigated this using native ion mobility mass spectrometry (nIM-MS).^63^ Their results provided an interpretation that partially reconciled the views of both groups. Specifically, they demonstrated that apo-IscU can adopt an intermediate conformational state in the absence of Zn^2+^, showing a wide range of charge distribution consistent with a partially disordered structure. By combining variable-temperature electrospray ionization with nIM-MS, they further characterized the cold- and heat-induced denaturation behavior of IscU in the presence or absence of Zn^2+^ or IscS. These experiments showed that Zn^2+^ binding shifts IscU toward a more compact conformation even under cold- and heat-denaturing conditions, whereas IscS binding does not induce the same degree of compaction. In addition, by incorporating collision-induced unfolding (CIU), they examined how cofactor or partner–protein binding affects the gas-phase stability of IscU conformers. Consistent with these findings, Bennett *et al.* reported similar results using native MS, although they did not explicitly describe the conformational ensemble in terms of an “intermediate” state.^64^ Their data also showed that Zn^2+^ binding promotes a more compact structure relative to apo-IscU. In addition, their study helped to define the mechanistic steps of the Fe–S cluster assembly during the formation of the IscU–IscS complex under anaerobic conditions.

Taken together, these considerations suggest that the views of Markley *et al.* and Pastore *et al.* are not mutually exclusive, but rather can be reconciled. In this context, the key issue then becomes whether the D-state has an appropriate functional role. We showed that IscU mutants that favor D-state exhibit increased binding preferences for HscA, implying the possible physiological importance of the D-state.^31,36^ Although further experimental validation is required, these observations indicate that the D-state may be considered a functionally relevant conformational ensemble that can be selectively utilized during specific stages of protein interactions and Fe–S cluster trafficking.

## III. FUTURE DIRECTIONS FOR ELUCIDATING HETEROGENEITY

IscU accommodates two contradictory functions: assembly/protection and efficient release of the [Fe–S] cluster. The structural metamorphosis of IscU, the interconversion between two structural states, and its dependence on the environment, such as the presence of metal ions and partner proteins, are consistent with these functions. Despite the functional importance of both conformations, only structural models of the S-state have been determined using x-ray crystallography, NMR spectroscopy, and cryo-EM.^25,39,41^ Structural characterization of the D-state of IscU, however, has only been partially achieved with NMR spectroscopy and native MS, likely reflecting the difficulty of capturing the highly dynamic D-state.^64^ In particular, the NMR spectra of apo-IscU exhibit signals from both the S- and D-states, leading to a substantial spectral overlap that hampered the selective characterization of the D-state. In addition, long-range nuclear Overhauser effect (NOE) signals for the D-state are difficult to observe because of their dynamic characteristics, thereby limiting the availability of the distance restraints required for atomistic structural characterization.^65^ To circumvent these challenges, several methodologies that have proven effective in IDP studies may be considered.

The application of high pressure is one of the mildest strategies for stabilizing unobservable minor states. High-pressure NMR spectroscopy (HP-NMR) incorporates this concept into NMR studies by modulating the pressure to stabilize low-population states and observing their corresponding NMR signals.^66^ Therefore, the D-state and other low-population intermediate states of IscU may be suitable targets for HP-NMR analysis. By stabilizing the D-state or intermediate conformations under high-pressure conditions, the reduction of the S-state population without introducing mutations or chemical additives may be possible. In addition, as discussed above, the cold-denatured states of IscU^57,67^ can be assessed through this approach. Our research group previously used HP-NMR to assess an S-state-stabilizing mutant, IscU (I8K).^68^ Under high-pressure conditions, the NMR spectra of this mutant resemble those of the D-state; however, certain residues exhibit increased sensitivity to pressure, thereby indicating differential structural responses.

Because the interconversion between the S- and D-states of IscU occurs on a relatively slow timescale, this slow exchange makes it difficult to fully characterize the kinetics and dynamics of the process using NMR alone. Single-molecule Förster resonance energy transfer (smFRET) is widely used for probing slow conformational exchange in proteins.^69^ This strategy is analogous, to some extent, to the paramagnetic relaxation enhancement (PRE) NMR method that we previously used to validate the interactions between the N-terminal and core regions of IscU.^36^

In addition, nIM-MS is a promising strategy for detecting multiple conformational states of IscU.^63,64^ This approach could also be extended to IscU-binding partners such as HscA and HscB. In particular, nIM-MS and CIU may provide methods for comparing the effects of different binding partners on the conformational ensemble, compactness, and stability of IscU. These analyses could help define the role of each partner protein in the IscU functional cycle.

Finally, computational approaches could be valuable alternatives for revealing the structural heterogeneity of IscU. Although artificial intelligence (AI)-based protein structure prediction methods, including AlphaFold2^70^ and RosettaFold,^71^ have greatly advanced structural biology, they still face challenges in the accurate prediction of proteins that adopt multiple conformations or contain intrinsically disordered regions.^72,73^ To address these limitations, approaches that combine multiple-sequence alignment (MSA) clustering with existing protein structure prediction tools have been proposed.^73^ By applying MSA clustering to IscU, structural models that represent a low population or D-state-like conformations may be obtainable. These models can serve as possible initial structures for subsequent molecular dynamics simulations and further refinements. Moreover, even without relying directly on AI-based structural prediction tools, co-evolutionary pattern analysis may provide a useful starting point for artificial mutation studies. For instance, statistical coupling analysis (SCA) of MSAs may reveal amino acid pairs that are responsible for the delicate balance sustaining IscU's heterogeneity.^74^ Indeed, our research group recently applied these approaches to investigate the transient structures and interactions of the N-terminal α-helix in IscU.^36^ Using AF-cluster analysis, we predicted the possible positions and conformations of this α-helix relative to the core region. In parallel, SCA of the IscU MSA was used to identify potential interaction sites within this region. These predictions were further examined using solution NMR methods, including PRE-NMR, and were consistent with the experimental results. Although this study relied mainly on predictions based on the S-state of IscU, similar approaches can be extended to generate possible models of the D-state conformational ensemble.

## IV. CONCLUSION

The structural heterogeneity of IscU extends beyond local flexibility and is intrinsically linked to its global conformational landscape—specifically, its metamorphic equilibrium—with direct functional implications for Fe–S cluster biosynthesis. However, the fundamental challenge lies not in the existence of this heterogeneity, but in its interpretation. IscU remains positioned between two competing models, described as either a metamorphic or marginally stable protein. This discrepancy arises from the limited understanding of the structural and functional identities of the D-state. In this context, we believe that the D-state may not be regarded as a passive or incidental conformation, but rather as a central unresolved element essential for understanding the structural heterogeneity of IscU. From this perspective, the elucidation of low-population transient conformational ensembles is a major direction for future research. Ultimately, resolving this issue will deepen our understanding of the structure–function relationship of IscU while providing critical insights into the molecular basis of Fe–S cluster homeostasis and its associated diseases.

## V. METHODS

Protein structures were retrieved from the Protein Data Bank (PDB) and analyzed using PyMOL.^75^ Structural alignment was performed using the built-in PyMOL algorithm, and the backbone RMSD values were calculated to assess the structural similarity among the IscU models under different experimental conditions. Previously reported raw NMR datasets generated by our research group were reprocessed and reanalyzed for comparative structural analysis.^36^ Multiple sequence alignments were generated using ClustalW^76^ and visualized using Jalview.^77^ A secondary structure alignment diagram was generated using an in-house Python3 script that included the pandas and matplotlib libraries.

## Data Availability

The data that support the findings of this study are available within the article.
